# Transitioning between medical equipment suppliers does not affect ACL reconstruction outcomes in a high‐volume setting

**DOI:** 10.1002/ksa.12775

**Published:** 2025-07-13

**Authors:** Dzan Rizvanovic, Anders Stålman, Christoffer von Essen

**Affiliations:** ^1^ Department of Molecular Medicine and Surgery, Stockholm Sports Trauma Research Center Karolinska Institutet Stockholm Sweden; ^2^ Capio Artro Clinic, Sophiahemmet Stockholm Sweden

**Keywords:** anterior cruciate ligament, KOOS, learning curve, operating time, revision

## Abstract

**Purpose:**

To investigate whether changing medical equipment suppliers in anterior cruciate ligament reconstruction (ACLR) influences surgical time, revision rates and subjective outcomes.

**Methods:**

A retrospective cohort study was conducted using data from the Swedish Knee Ligament Registry (SKLR). On 1 September 2016, Capio Artro Clinic, Stockholm, Sweden, changed its supplier for all medical equipment, from Smith & Nephew (SN) to Arthrex (AR). Patients who underwent primary ACLR during the preceding year (from 1 September 2015) formed group SN and were compared with patients operated between 1 September 2016 and 1 September 2017, divided into three consecutive 4‐month cohorts (AR1–3) for temporal comparison. Primary outcome was surgical time. Secondary outcomes were revision rates at 2 and 5 years, and the aggregate Knee injury and Osteoarthritis Outcome Score (KOOS_4_) at 2 years using thresholds for minimal important change (MIC), Patient Acceptable Symptom State (PASS), treatment failure (TF) and perioperative complications.

**Results:**

A total of 1175 patients underwent a primary ACLR during the study period. Group SN included 521 patients, and Groups AR1–3, 654. Of the 521 patients in Group SN, 488 (93.7%) had surgical time recorded, with a median of 61 (interquartile range [IQR]: 50–73) min. In Groups AR1–3, 560 patients (85.6%) had surgical time recorded, with median times of 63 (IQR: 52–78), 60 (IQR: 50–70) and 60 (IQR: 50–70) min, respectively, showing no significant differences. Of the included patients, 573 (48.8%) completed KOOS at the 2‐year follow‐up, with no significant differences between groups regarding MIC, PASS or TF. Within 2 years, 31 patients (2.6%) underwent revision ACLR, and after 5 years, an additional 21 patients, totalling 52 (4.4%), with no significant group differences.

**Conclusion:**

Experienced ACL surgeons can transition between suppliers with acceptable outcomes, comparable surgical times and low complication rates.

**Level of Evidence:**

Level III.

**Abbreviations**
ACL: anterior cruciate ligament
ACLR: anterior cruciate ligament reconstruction
AMP: anteromedial portal
AR1‐3: groups based on surgery date after supplier change
BPTB: bone–patellar tendon–bone
HT: hamstring tendon
IQR: interquartile range
KOOS: Knee injury and Osteoarthritis Outcome Score
KOOS_4_
: mean score of the KOOS Pain, Symptoms, Sports/Rec and QoL subscales
MIC: minimal important change
PASS: Patient Acceptable Symptom State
QT: quadriceps tendon
SKLR: Swedish Knee Ligament Registry
SN: group prior to supplier change
TF: treatment failure

## INTRODUCTION

Anterior cruciate ligament (ACL) reconstruction (ACLR) is widely accepted as the standard treatment option for active individuals with functional instability of the knee joint due to ACL injury. Developing proficiency in ACLR comes with a learning curve [13, 27]. Studies have shown that experienced surgeons perform ACLRs with shorter operating times [19], more anatomical graft placement [6] and utilize a greater variety of autografts [20]. Additionally, higher surgical volumes have been associated with improved clinical outcomes and patient satisfaction, reduced readmission and complication rates, higher rates of meniscal repair and lower total costs [14, 19, 21, 25, 27].

Over time, techniques used for ACLR have evolved [3, 11]. Several companies specialize in orthopaedic products used for ACLR. Although these products are often similar and utilize comparable techniques, they each have their pearls and pitfalls, making them not completely interchangeable [7, 16]. There can be many reasons for changing suppliers of medical products, such as surgeon preference, procurement and financial considerations. When a change is made, it is important to ensure that the outcomes and complication rates with the new technique are acceptable and comparable to the standard of care. While some orthopaedic studies have focused on the learning curve of different techniques [13, 27], the impact of changing manufacturers on outcomes following ACL reconstruction remains unclear.

The purpose of this study was to evaluate whether experienced ACL surgeons could transition from one company's product to another, comparing surgical time, revision rates, and subjective outcomes during the first year with those of the last year. It was hypothesized that differences would be observed in the studied parameters during the first year.

## METHODS

Ethical approval was obtained from the Regional Ethical Review Board (2016/1613‐31/2).

A retrospective registry‐based cohort study was conducted using data from the Swedish Knee Ligament Registry (SKLR). The SKLR is a nationwide database, initiated in January 2005, that uses a web‐based protocol for registration. Data are collected prospectively, and the surgeon registers all surgical procedures, including meniscal surgeries and chondral treatments as well as perioperative complications. The SKLR contains information on graft type, fixation techniques, surgical time and time from injury to surgery. It is estimated that there is at least a 90% coverage of all ACL surgeries in Sweden [12]. The patients register information about their lifestyle and fill in the Knee injury and Osteoarthritis Outcome Score (KOOS) [23] pre‐injury and at 6,12 and 24 months.

When the patient undergoes revision surgery or reoperation by any means, the surgeon registers this as separate entries, and the event is correlated to the index ACL reconstruction. Extracted data from the database are anonymous, and investigators only have access to unidentifiable patient data.

Capio Artro Clinic, Stockholm, Sweden, is considered a high‐volume centre, performing approximately 800 primary ACLRs and revisions annually between 2015 and 2017 [20]. All ACLRs were performed by experienced surgeons defined as sports medicine fellowship‐trained. On 1 September 2016, the clinic changed its supplier for all arthroscopy‐ and ACLR‐related medical equipment from Smith & Nephew to Arthrex, including imaging systems, arthroscopy accessories, surgical instruments and fixation devices (except for tibial fixation). To analyze this transition, patients who were registered for primary index ACLR during the preceding year (from 1 September 2015) (group SN) were compared to patients registered for primary ACLR between 1 September 2016 and 1 September 2017 (Groups AR1–3). Groups AR1–3 were divided into 4‐month blocks to illustrate evolvement over time and to illustrate the first ACLRs for the surgeons (AR1: Sep–Dec 2016; AR2: Jan–Apr 2017; and AR3: May–Aug 2017, including 1 September 2017). The surgeons received relevant information and performed basic testing on models prior to clinical use.

Patient inclusion criteria included age 16 years or older and having undergone a primary ACLR at Capio Artro Clinic, Stockholm, Sweden, between 1 September 2015 and 1 September 2017. The exclusion criteria were prior ACLR on the affected knee, any concomitant ligament injury of the affected knee that needed to be addressed, tendon/vascular/nerve injuries, and if the ACLR was conducted with a graft other than hamstring tendon (HT), quadriceps tendon (QT) or bone–patellar tendon–bone (BPTB) autograft.

### Surgical technique

All ACLRs were performed under general anaesthesia, and apart from the implants, the surgical procedure was identical for both groups. After an initial diagnostic arthroscopy, the patients underwent an arthroscopic single bundle ACLR with an HT, QT or BPTB autograft. An anteromedial portal (AMP) technique was used. If the ACLRs were performed with HT grafts, the semitendinosus tendon was harvested and prepared as a quadruple graft and if insufficient in length or diameter (<8 mm), the gracilis tendon was harvested as well. The BPTB graft was harvested as the central third of the patellar tendon with two bone blocks. The QT was harvested with a bone block. For Group SN, an Endobutton or Endobutton BTB (Smith & Nephew) fixation device was routinely used for the femoral side and on the tibial side Ethibond no. 2 sutures (Ethicon) were tied over a 4.5‐mm AO bicortical screw with a washer (Smith & Nephew) as a post. Group AR1–3 used an adjustable loop TightRope™ or TightRope BTB™ (Arthrex, Inc.) and armed with Ethibond no. 2 sutures (Ethicon) tied over a 4.5‐mm AO bicortical screw with a washer (Smith & Nephew). All patients were instructed to participate in a standardized post‐surgical rehabilitation plan. The patients were allowed to return to sports 9 months after ACLR at the earliest if the isokinetic knee muscle strength and single‐leg‐hop test criteria (Limb Symmetry Index ≥90%) were met.

### Outcome measures

The primary outcome of interest was surgical time for the ACLR. Secondary outcomes were revision ACLR rates at 2 and 5 years, KOOS_4_ at 2 years and perioperative complications, which were considered relevant by the surgeon.

The KOOS comprises 42 items divided into five subscales: pain, symptoms, activities of daily living (ADL), sport and recreation (Sport/Rec) and quality of life (QoL). Each item is answered on a 5‐point Likert scale, and then the items of each subscale are transformed to a subscale score ranging from 0 (*worst*) to 100 (*best*). This study analyzed KOOS_4_ [15], thus excluding the subscale ADL. Threshold values for minimal important change (MIC, ≥9), Patient Acceptable Symptom State (PASS, ≥79) and treatment failure (TF, ≤42) were applied to determine the results of clinical importance [9, 10, 22].

### Statistical analyses

SPSS Statistics Version 29 (IBM Corp) was used to conduct all analyses. Descriptive statistics were used to summarize all the variables. Median (interquartile range [IQR]) was reported for nonnormally distributed continuous variables, and the Kruskal–Wallis test was used for between‐group comparisons. Counts and percentages were presented for categorical variables, and the chi‐square test was used for between‐group comparisons. Significant between‐group differences were further evaluated with pairwise group comparisons using the Mann–Whitney *U* test (continuous variables) and the chi‐square or Fisher exact test (categorical variables). A post hoc correction was conducted for pairwise comparisons (Bonferroni‐adjusted *p* values). Statistical significance was set at a *p* value <0.05 (two‐tailed) in all analyses. For the main outcome measure, surgical time, a post hoc power analysis was conducted using G*Power version 3.1 (effect size *f* = 0.25, *α* = 0.05, four groups, total *N* = 1175), which yielded a power of 1.00, indicating that the study was more than adequately powered to detect a moderate difference between groups.

## RESULTS

A total of 1175 patients underwent ACLR during the study period. Group SN consisted of 521 patients, and Group AR1–3 of 654 patients (234/222/198). A total of 19 different surgeons performed the ACLRs. Patient characteristics are presented in Table 1. There was a significantly higher proportion of meniscal sutures of the medial meniscus tears in group AR3 compared to Group SN (60.8% vs. 32.4%). No other differences were found regarding meniscal repair or injuries. Altogether, 500 patients had meniscal tears, either on the medial, the lateral or both. A total of 571 meniscal tears were diagnosed, and 177 (20.5%) of these tears were repaired.

**Table 1:** Patient characteristics.

	Group SN (*n* = 521)	Group AR1 (*n* = 234)	Group AR2 (*n* = 222)	Group AR3 (*n* = 198)	*p*	Test between‐groups *p* value, Bonferroni‐adjusted		
						SN vs. AR1	SN vs. AR2	SN vs. AR3
Age, years	30.0 (22.0–40.0)	29 (21.0–40.0)	29 (21.0–40.0)	27.5 (21.0–41.0)	n.s.			
Women, *n* (%)	233 (44.7)	118 (50.4)	110 (49.5)	98 (49.5)	n.s.			
Injury at pivoting sport, *n* (%)	242 (46.4)	115 (49.1)	108 (48.6)	72 (36.4)	n.s.			
Meniscal injury, *n* a (%)	246 (47.2)	96 (41.0)	80 (36.0)	78 (39.4)	0.023	n.s.	0.005	n.s.
Medial meniscus injury	145 (58.9)	59 (61.4)	57 (71.3)	51 (65.4)	n.s.			
Medial meniscus suture	47 (32.4)	19 (32.2)	22 (38.6)	31 (60.8)	0.003	n.s.	n.s.	<0.001
Lateral meniscus injury	135 (54.9)	54 (56.3)	32 (40.0)	38 (48.7)	n.s.			
Lateral meniscus suture	27 (20.0)	13 (24.1)	8 (25.0)	10 (26.3)	n.s.			
Cartilage injury	68 (13.1)	33 (14.1)	35 (15.8)	37 (18.7)	n.s.			
Cartilage surgery	19 (27.9)	10 (30.3)	15 (42.9)	17 (45.9)	n.s.			
ACL graft					<0.001	<0.001	n.s.	<0.001
BTB	36 (6.9)	39 (16.7)	25 (11.3)	33 (16.7)				
HT	478 (91.7)	187 (79.9)	193 (86.9)	160 (80.8)				
QT	7 (1.3)	8 (3.4)	4 (1.8)	5 (2.5)				

Abbreviations: ACL, anterior cruciate ligament; AR1–3, groups based on surgery date after supplier change; BTB, patella‐bone tendon‐bone graft; HT, hamstring tendon; QT, quadriceps tendon; SN, group prior to supplier change.

a Number of patients.

Of the 521 patients in Group SN, 488 patients (93.7%) had surgical time recorded, with a median of 61 (IQR: 50–73) min. In Group AR1–3, 560 patients (85.6%) had surgical time recorded, and the median surgical time was 63 min (IQR: 52–78), 60 (50–70) min and 60 (50–70) min, respectively, with no significant difference between the periods or the groups (Table 2). There was also no difference when the groups were compared separately.

**Table 2:** Reported surgical time and complications between the groups.

	Group SN (*n* = 521)	Group AR1 (*n* = 234)	Group AR2 (*n* = 222)	Group AR3 (*n* = 198)	*p*	Test between‐groups *p* value, Bonferroni‐adjusted		
						SN vs. AR1	SN vs. AR2	SN vs. AR3
Surgical time, min	61.0 (50.25–73.0)	62.5 (52.0–78.0)	60.0 (50.0–70.0)	60.0 (50.0–70.3)	n.s.			
Missing	33 (6.3)	22 (9.4)	36 (16.2)	36 (18.1)				
Perioperative complications	1 (0.2)	1 (0.4)	1 (0.5)	6 (3.0)	0.001	n.s.	n.s.	<0.001
Revision <2 years	16 (3.1)	5 (2.1)	5 (2.3)	5 (2.5)	n.s.			
Revision <5 years	28 (5.4)	9 (3.8)	7 (3.2)	8 (4.0)	n.s.			

Abbreviations: AR1–3, groups based on surgery date after supplier change; SN, group prior to supplier change.

There were 31 patients (2.6%) who underwent revision ACLR within 2 years of primary ACLR (Table 3). After 5 years, an additional 21 patients had undergone revision ACLR, making a total of 52 patients (4.4%). No significant differences were found between the groups.

**Table 3:** Percentage of patients meeting criteria of MIC, PASS and TF of the aggregate Knee injury and Osteoarthritis Outcome Score (KOOS_4_).

	Group SN (*n* = 521)	Group AR1 (*n* = 234)	Group AR2 (*n* = 222)	Group AR3 (*n* = 198)	*p*	Test between‐groups *p* value, Bonferroni‐adjusted		
						SN vs. AR1	SN vs. AR2	SN vs. AR3
Preoperative KOOS_4_, median (IQR)	59.5 (46.6–69.9)	62.0 (48.1–79.5)	62.0 (49.4–76.4)	65.3 (45.4–100.0)	0.003	0.048	0.040	0.012
KOOS_4_ at 2 years, median (IQR)	81.1 (67.8–90.7)	80.1 (66.1–88.4)	80.2 (64.3–88.4)	79.6 (65.4–93.3)	n.s.			
KOOS_4_ at 2 years, *n* (%)	279 (53.5)	93 (39.7)	114 (51.4)	88 (44.4)	0.002	<0.001	n.s.	n.s.
PASS, *n* (%)	146 (54.1)	48 (52.2)	63 (55.8)	44 (52.5)	n.s.			
TF, *n* (%)	42 (15.6)	18 (19.6)	17 (15.0)	11 (12.9)	n.s.			
MIC, *n* (%)	192 (72.2)	59 (64.8)	74 (69.2)	49 (59.0)	n.s.			

Abbreviations: AR1–3, groups based on surgery date after supplier change; IQR, interquartile range; MIC, minimal important change (≥9); PASS, patient acceptable symptom state (≥79); SN, group prior to supplier change; TF, treatment failure (≤42).

The rates of perioperative complications were significantly higher (*p* = 0.001) for Group AR3. However, no other differences were found during the earlier phases. The following complications were reported among the different groups. In Group SN, there was one incident of a punctured blood pressure cuff. In Group AR1, one case of a TightRope cutting through the bone block was reported. Group AR2 had one case of severe bradycardia requiring hospital admission and observation. In Group AR3, the reported complications included two incidents of incorrectly packed instruments from the sterilization unit, one incident of a graft being dropped on the floor, a bone block getting stuck, a TightRope with an extender failing to flip, and a TightRope button pulling through the skin.

Of the included patients, 570 patients (48.5%) completed KOOS at a 2‐year follow‐up. In comparison between the groups, there was no difference in MIC, PASS or TF (Table 3).

## DISCUSSION

The most important finding of the study was the absence of significant differences between the groups in objective or subjective measures following the transition to new implants, instrumentation and techniques for ACLR. These findings suggest that experienced surgeons can adapt to new equipment without compromising surgical time, revision rates or patient‐reported outcomes. Although a statistically significant increase in reported perioperative complications was observed in the AR3 group compared to the other groups, the overall number of complications remained low (six cases in AR3 vs. one to two cases in the other groups). Due to the limited occurrence, it is uncertain whether this difference is clinically relevant, and no negative impact on overall outcomes was observed.

When transitioning to new surgical techniques or instrumentation, a learning curve is often anticipated. However, the present study's findings indicate that experienced surgeons were able to maintain their performance standards without observable adaptation periods. This aligns with findings from Snow and Stanish [26], who demonstrated that experienced ACL surgeons could transition from a transtibial single‐bundle technique to an AMP double‐bundle technique with accuracy within the first 10 cases. Conversely, Luthringer et al. [13] suggested that mastering independent AMP drilling required a learning curve of at least 32 cases, and Hohmann et al. [5] reported significant improvements in tunnel placement after 100 ACLRs. The differences in these findings could be attributed to the complexity of the surgical techniques being assessed or the experience level of the surgeons involved. In the present study, the absence of a measurable difference may be attributed to the high level of experience among the participating surgeons.

The lack of significant differences in surgical time is noteworthy. Previous studies, such as Talme et al. [27], reported that surgical time decreases markedly during the first 20 reconstructions performed by inexperienced surgeons. However, this study exclusively involved experienced arthroscopic surgeons, likely accounting for the uniformity in surgical time across groups. Interestingly, the AR3 group demonstrated a significantly higher proportion of meniscal repairs compared to the SN group, yet this did not prolong surgical time. Additionally, variations in graft types, with a higher frequency of BTB grafts in AR1 and AR3 groups, also did not influence surgical time. These findings are consistent with those of Huyke‐Hernández et al. [8], who reported no difference in surgical time across different autografts.

Subjective outcomes, measured by the KOOS, revealed no significant differences between the groups at the 2‐year follow‐up. The consistency of KOOS outcomes aligns with the findings of Rizvanovic et al. [19], who highlighted the advantages of having ACLR performed by experienced surgeons. Despite differences in graft choices across groups, the rates of MIC, PASS and TF were consistent and comparable to those reported in the literature [4, 17, 19].

Although the rate of perioperative complications was statistically significantly higher in the AR3 group, the difference remained clinically negligible, especially considering that only two of these complications were directly related to the device used. Additionally, it is important to highlight that the reported complication rates rely on surgeon self‐reporting in the SKLR, which may result in underreporting or inconsistent documentation. Therefore, given the overall low number of perioperative complications and potential reporting biases, these findings should be interpreted cautiously.

## STRENGTHS AND LIMITATIONS

The primary strength of this study lies in its large sample size. The use of the SKLR enabled the identification of revisions and outcomes across the entire country, enhancing the robustness of the findings. Furthermore, all surgeries were conducted at the same institution, ensuring consistency in surgical technique and post‐operative rehabilitation protocols.

However, the study also has limitations. All ACLRs were performed at a high‐volume clinic, which may limit the generalizability of our results beyond similar environments. Its retrospective nature inherently limits causal inferences. Additionally, the inclusion of different surgeons, concomitant injuries, and graft types introduces potential confounding factors that may influence both surgical time and outcomes of ACLR [1, 15]. The loss to follow‐up in KOOS data, with approximately half of the patients not responding at 2 years, represents another limitation. However, the follow‐up rate was consistent with other registry‐based studies [2, 24]. While a significant difference in response rates was observed between the SN and AR1 groups, this is unlikely to have impacted the results. A previous study from the SKLR reported lower response rates among younger patients, male patients, and those with lower socioeconomic status, but found no clinically relevant differences in KOOS subscales between responders and non‐responders [18]. Finally, the reliance on self‐reported data for perioperative complications may have resulted in underreporting or subjective biases.

## CONCLUSION

The present study shows that it is possible for an experienced ACL surgeon to convert from one medical company's equipment to another with acceptable outcomes, surgical time and complications within the first patients.

## AUTHOR CONTRIBUTIONS

All authors contributed to the conception and design of the study. Dzan Rizvanovic was responsible for data extraction and analysis from SKLR. Christoffer von Essen wrote the initial draft of the manuscript. Dzan Rizvanovic and Anders Stålman critically revised the manuscript. All authors had full access to the data, participated in interpretation of the findings, and approved the final version. Anders Stålman is the study guarantor.

## CONFLICT OF INTEREST STATEMENT

Anders Stålman is a member of the steering committee of the Swedish Knee Ligament Registry (SKLR). The remaining authors declare no conflicts of interest.

## ETHICS STATEMENT

The study was approved by the Regional Ethical Review Board in Stockholm (2016/1613‐31/2).

## Data Availability

The data supporting the findings of this study are available from the corresponding author upon reasonable request and approval from the Swedish Knee Ligament Registry committee.
